# *In Silico* Screening of Nonsteroidal Anti-Inflammatory Drugs and Their Combined Action on Prostaglandin H Synthase-1

**DOI:** 10.3390/ph3072059

**Published:** 2010-07-02

**Authors:** Alexey Goltsov, Galina Lebedeva, Ian Humphery-Smith, Gregory Goltsov, Oleg Demin, Igor Goryanin

**Affiliations:** 1 Centre for Research in Informatics and Systems Pathology, School of Contemporary Sciences, University of Abertay Dundee, Dundee, DD1 1HG, UK; 2 Centre for Systems Biology at Edinburgh, University of Edinburgh, The King’s Buildings, Edinburgh, EH9 3JZ, UK; E-Mail: glebedev@staffmail.ed.ac.uk; 3 Deomed, 21 Emblehope Drive, Gosforth, Newcastle-upon-Tyne, NE3 4RW, UK; E-Mail: ianhs@hotmail.com; 4 School of Informatics, University of Edinburgh, Edinburgh, EH8 9AB, UK; E-Mails: g.goltsov@sms.ed.ac.uk (G.G.); goryanin@inf.ed.ac.uk (I.G.); 5 Institute for Systems Biology SPb, Sankt-Peterburg, Toresa 80-48, Russia; E-Mail: demin@insysbio.ru (O.D.); 6 A.N. Belozersky Institute of Physico-Chemical Biology, Moscow State University, Moscow, 119992, Russia

**Keywords:** kinetic modeling, COX-1,2, NSAID, aspirin resistance, NSAID combination

## Abstract

The detailed kinetic model of Prostaglandin H Synthase-1 (PGHS-1) was applied to *in silico* screening of dose-dependencies for the different types of nonsteroidal anti-inflammatory drugs (NSAIDs), such as: reversible/irreversible, nonselective/selective to PGHS-1/PGHS-2 and time dependent/independent inhibitors (aspirin, ibuprofen, celecoxib, *etc.*) The computational screening has shown a significant variability in the IC_50_s of the same drug, depending on different *in vitro* and *in vivo* experimental conditions. To study this high heterogeneity in the inhibitory effects of NSAIDs, we have developed an *in silico* approach to evaluate NSAID action on targets under different PGHS-1 microenvironmental conditions, such as arachidonic acid, reducing cofactor, and peroxide concentrations. The designed technique permits translating the drug IC_50_, obtained in one experimental setting to another, and predicts *in vivo* inhibitory effects based on the relevant *in vitro* data. For the aspirin case, we elucidated the mechanism underlying the enhancement and reduction (aspirin resistance) of its efficacy, depending on PGHS-1 microenvironment in *in vitro*/*in vivo* experimental settings. We also present the results of the *in silico* screening of the combined action of sets of two NSAIDs (aspirin with ibuprofen, aspirin with celecoxib), and study the mechanism of the experimentally observed effect of the suppression of aspirin-mediated PGHS-1 inhibition by selective and nonselective NSAIDs. Furthermore, we discuss the applications of the obtained results to the problems of standardization of NSAID test assay, dependence of the NSAID efficacy on cellular environment of PGHS-1, drug resistance, and NSAID combination therapy.

## 1. Introduction

The key pharmacological targets of NSAIDs are two isoforms of Prostaglandin H Synthase (PGHS) –PGHS-1 and PGHS-2 (also referred to as cyclooxygenase-1,2, COX-1,2) [1,2]. PGHS is a complex bifunctional, membrane-bound enzyme, which has two catalytic sites: a cyclooxygenase site (COX-site), in which the substrate, arachidonic acid, AA is oxygenized to the intermediate product, prostaglandin G_2_ (PGG_2_), and a peroxidase site (POX-site), in which PGG_2_ is reduced to the final product, prostaglandin H_2_ (PGH_2_) [2]. 

Extensive studies of the enzyme have shown that PGHS catalysis is a complex phenomenon, involving the formation of multiple enzyme intermediates, redox transformation of key catalytic components by intraprotein electron transport, cooperative interaction of POX and COX activities, self-inactivation, and activation threshold of the enzyme. Despite significant progress in the study of PGHS over more than 30 years, many features of the enzyme catalysis are still to be clarified [3]. 

It is suggested that the complex properties of the enzyme determine strict regulation of the synthesis of prostaglandin H_2_, a precursor of signaling prostanoid molecules such as prostacyclin, thromboxane, *etc.* [3,4]. Furthermore, it has been shown that the specific properties of PGHS manifest themselves significantly in the inhibitory effects of NSAIDs [1,5,6]. In particular, the structure of the catalytic COX-site, comprised of a hydrophobic channel for AA binding, defines several classes of NSAIDs that differ from one another in the mechanisms of binding with this channel: irreversible (aspirin), reversible (naproxen, diclofenac), time-dependent (indomethacin, celecoxib), time-independent (ibuprofen, naproxen), and selective to COX-1 or COX-2 NSAIDs [7,8,9,10].

The other indication of the complex dynamics of the interaction of PGHS with NSAIDs was observed in the experimental screening of NSAID dose-dependencies, drug IC_50_s, and selectivity [7,9,11,12,13,14,15,16,17]. These experimental data showed that drug effects depend dramatically on the experimental assays and microenvironment of PGHS-1. The experimental study of NSAID inhibition effects is commonly performed with the use of different assays, among them: purified PGHS-1,2 [7,11,12,13], intact cells (platelet, endothelial cells and others) [13,14,15,16], and human whole blood assay (WBA) [9,17]. The key properties of NSAIDs, such as dose-dependencies, IC_50_s, their basic types, and mechanism of action were mainly characterised in *in vitro* experimental screenings with the use of cell-free preparations of PGHS-1,2 [7,11,12]. 

The experimental results showed that IC_50_s and selectivity values obtained for the same NSAID in different experimental settings, may differ from each other by up to two orders of magnitude [10,14]. Moreover, two drugs may produce equivalent effects in one assay and show different effects under other conditions [17]. As a result of experimental studies, it was suggested that the key factor causing the variation in NSAID effects, is the difference between *in vitro* experimental conditions and the intracellular microenvironments in various cells [17]. The observed variation of drug IC_50_ complicates the comparison of the different drug efficacies. This effect also makes difficult to translate the results, obtained in *in vitro*, to *in vivo* conditions [10,14]. Furthermore, discrepancy and uncertainty in IC_50_ values and selectivity of some NSAIDs, lead to uncertainty in the prediction of the side effects of the drugs, which are determined by their selectivity to PGHS-1/PGHS-2 [18]. 

On one hand this problem points to the need for a standardization of the drug testing assays which would permit the comparison of different compounds [10,17]. On the other hand, the observed discrepancy of the IC_50_s indicates the large sensitivity of NSAIDs to the PGHS environment and cell types. The variation in the sensitivity of NSAIDs observed in *in vivo* assays is likely to exhibit itself on the organism level, in the form of variability of the response of different individuals to the same drug. For example, clinical studies show the heterogeneity in the suppression of platelet PGHS-1 activity by aspirin, and low response to aspirin in patients with coronary heart disease *versus* a healthy cohort [19,20]. Low aspirin responsiveness or aspirin resistance [21] is assumed to be caused by hyperactive platelets due to the local high concentration of AA at the site of vascular injury [22,23]. 

Another clinical observation of different individuals’ response to NSAIDs relates to the variation in cardiovascular side effects, found among individuals taking PGHS-2 inhibitors. The reasons for aspirin resistance and an individual’s risk of cardiovascular complications remain an unanswered question [20,24,25].

To analyse the variation of NSAID effects, we have developed an *in silico* experimental method to screen the NSAID action in the different microenvironment of PGHS-1. The computational simulation method is based on the detailed kinetic model of drug target and NSAIDs interaction with PGHS-1. The developed technique allows studying what influence a drug target’s microenvironment can have on the resulting drug effects.

In this work we demonstrate the abilities of the *in silico* screening method through its application to three NSAIDs: aspirin, ibuprofen, and celecoxib. These drugs represent three different classes of NSAIDs: aspirin is an irreversible, time-dependent traditional NSAID with preferential selectivity to PGHS-1 [13,16]; ibuprofen is a reversible, time-independent, PGHS-1 selective inhibitor, whereas celecoxib belongs to a new generation of PGHS-2 selective inhibitors with reversible time-independent binding to PGHS-1 and time-dependent binding to PGHS-2 [7]. Based on the calculations of the dose- dependencies for these drugs, we discuss the mechanisms of IC_50_ dependence on different PGHS-1 environmental conditions.

We also applied the computational screening method to study the problem of the combined action of two NSAIDs on PGHS-1. Administration of combined NSAID therapy is recommended for patients who take low-dose aspirin to reduce the risk of recurrent myocardial infarction or a stroke, while using NSAIDs such as celecoxib, ibuprofen, naproxen, *etc*. for arthritis pain [26,27]. Certain clinical data indicates an increased risk of cardiovascular complications in such patients, as compared to the patients taking aspirin alone [26,27]. The extensive studies of the interaction between aspirin and other NSAIDs *in vitro* and in clinic trials showed the ability of NSAIDs, such as celecoxib or naproxen, to suppress the aspirin-induced inhibitory effect on PGHS-1, their capabilities to prevent platelet aggregation, and blocking arteries [11,28].

To study the effect of NSAID interaction, we carried out *in silico* experiments with the combinations of aspirin and ibuprofen, as well as aspirin and celecoxib, in the different environmental conditions of PGHS-1. On the basis of the obtained results, we discuss the suppression mechanism of the aspirin-induced inhibitory effect on PGHS-1 by celecoxib and ibuprofen [11], and analyse the conditions which influence this effect.

## 2. Kinetic Model of Inhibitory Action of NSAIDs on PGHS-1

We have developed a kinetic model of the NSAID inhibition effect on PGHS-1, which includes detailed submodels of PGHS-1 catalysis and the enzyme interaction with NSAID [29]. Below we describe in brief our approach to modeling the complex functioning of PGHS-1 and the basic assumptions used on describing the action of different NSAIDs on PGHS-1. 

### 2.1. Detailed kinetic model of PGHS-1

**Figure 1:**
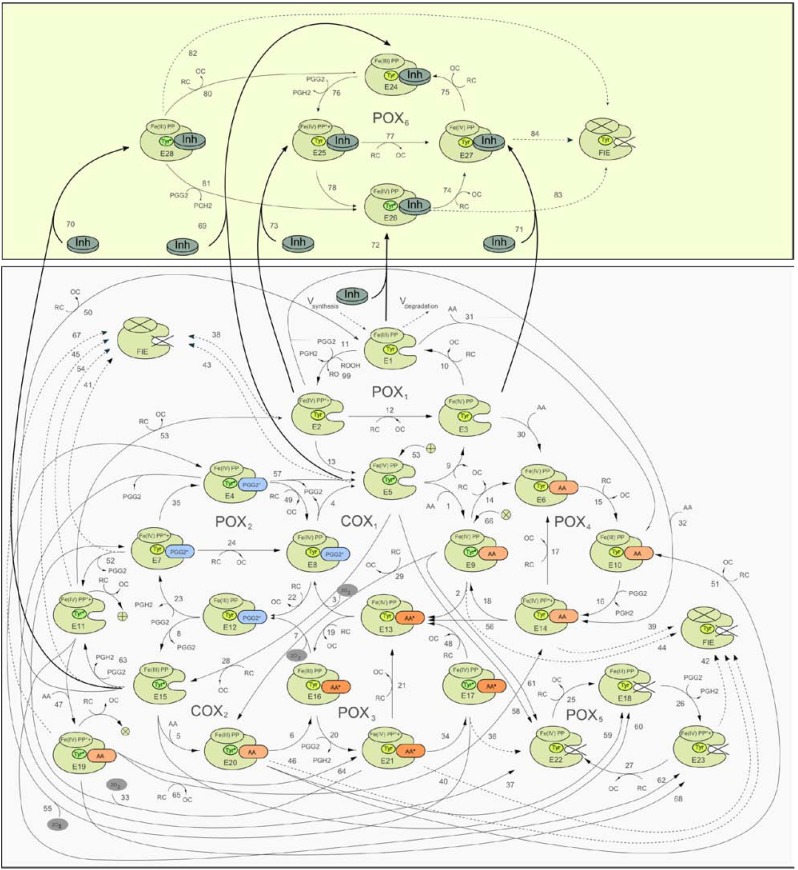
Scheme of catalytic cycle of PGHS with consideration of the enzyme inhibition by NSAID. Bottom panel—catalytic cycle of PGHS; top panel—scheme of NSAID (Inh) binding with the enzyme (thick lines); AA—arachidonic acid; RC—reducing cosubstrate; OC—oxidized RC; O_2_—oxygen; PGG_2_, PGH_2_—prostaglandin G_2_ and H_2_, respectively; E_i_—enzyme states; Fe(III) and F(IV)—ferric and oxiferryl states of heme iron; PP and PP*+—protoporphyrin group in ground and radical cation states; Tyr, Tyr*—Tyrosine 385 in ground and radical states; FIE—full inactivated enzyme state; cross—enzyme inactivated sites; dashed lines—reactions of self-inactivation of PGHS.

The developed scheme of the PGHS-1 catalytic cycle, including the interaction of the inhibitor with the enzyme, is shown in Figure 1. The bottom panel in Figure 1 shows the detailed catalytic cycle of PGHS-1 [29]. The upper panel presents the kinetic scheme of the interaction of inhibitor with PGHS-1. Below we describe in brief the kinetic model of PGHS-1 catalysis used in this work. 

The catalytic cycle proposed by us [29] is a further development of the approach based on the Branched Chain Mechanism of PGHS catalysis proposed by Dietz *et al*. [30] and elaborated further by Kulmacz *et al*. [31,32,33,34]. We considered the PGHS catalytic cycle as a system of elementary reactions between enzyme intermediates (E_i_, i=1,…,23), arachidonic acid (AA), reducing cosubstrate (RC), intermediate, PGG_2_ and final, PGH_2_ products. The full catalytic cycle of PGHS was presented as a composition of eight interlinked cycles: five of them corresponding to peroxidase activity (POX_i_, i=1,…,5), the other three—to cyclooxygenase activity (COX_i_, i=1,2,3) of the enzyme.

Two interconnected cycles, COX_1_ (reactions 1–4) and POX_1_ (reactions 9–13) correspond to the classical Branched Chain Mechanism of PGHS catalysis [30]. According to this mechanism, PGHS-1 catalysis is initiated in the POX-site, and its first step is the reaction of the resting enzyme [Fe(III), PP] (E1) with PGG_2,_ and production of PGH_2_ (reaction 11), followed by leaving the heme prosthetic group in the state [Fe(IV), PP*+] (E2, Intermediate I). The E2 state can be converted back to the resting state E1 by two consecutive reactions with an external reducing cosubstrate, RC, with a release of oxidised cosubstrate, OC (reactions 10 and 12). Alternatively, the E2 state can undergo a one-electron internal reduction via intramolecular electron transfer from the tyrosine 385 residue (Tyr385) to a protoporphyrin group (reaction 13), resulting in the formation of the E5 state, [Fe(IV), PP, Tyr^*^] containing the Tyr385* radical and a ferryl heme (Intermediate II). The E5 state can also complete the POX cycle and return to the resting state E1 via reactions 9 and 10 with cosubstrate. According to the Branched Chain Mechanism, the presence of the Tyr385* radical is ultimately required for the activation of AA oxidation reaction in the COX-site [1,35]. Thus the intermediate state E5 potentially serves as a starting point for the further cascade of the cyclooxygenase catalysis.

Reaction 1 describes a reversible binding of AA with the enzyme state E5, resulting in the formation of the state [Fe(IV), PP, Tyr^*^, AA] (E9), containing AA in the COX-site. Reaction 2 corresponds to the abstraction of a hydrogen atom from AA by Tyr385* radical and formation of the E13 enzyme state containing AA* radical in the COX-site, [Fe(IV), PP, Tyr, AA*]. This AA^*^ radical then reacts with two oxygen molecules and the intermediate product, PGG_2_^*^ radical, is produced (reaction 3). The final step of the COX_1_ cycle in our model is the irreversible reaction 4, which describes the process of E5 state regeneration with Tyr385^*^ radical, accompanied by the release of the intermediate product, PGG_2_ from the COX-site [1,2]. 

Taking into account that POX activity of PGHS proceeds independently from COX catalysis [2,35], we considered in our model the possibility of several additional POX_i_ and COX_i_ catalytic cycles, originating from the intermediate states involved in POX_1_ and COX_1_ cycles.

For example, the states of COX_2_ cycle originates from the states of COX_1_ cycle due to redox reactions with reducing cosubstrate, RC (reactions 19, 22, 28, and 29).

Similarly, POX_2_, POX_3_, and POX_4_ cycles result from states E8, E9, and E13 respectively, which can participate in peroxidase reactions. These cycles differ from POX_1_ by the presence of AA, AA* and PGG*in the COX-site.

The third COX_3_ cycle, though not explicitly shown in the scheme in Figure 1, can be defined as a cycle, formed by the enzyme states E7, E11, E19, and E21, involving reactions 47, 64, 33, and 53, respectively. Cycle POX_5_ was introduced to the model to account for the remaining POX activity of the enzyme, when COX-site is inactivated.

In the model we have taken into account the process of irreversible self-inactivation of the enzyme during catalysis [36,37,38]. Self-inactivation is assumed to be caused by radical damage of the relevant catalytic domains of the enzyme, occurring when it is functioning [3]. As a result of self-inactivation, the enzyme works for approximately 20 seconds. In the model [29], we have considered self-inactivation of COX and POX activities separately, distinguishing between the inactivation, which originates from the enzyme intermediates with different redox states of heme group, and marked out the inactivation processes proceeding from the states with one or two radicals in the catalytic domains [38]. Inactivation stages are shown as the dashed arrows in the scheme in Figure 1. 

We took into account the heterodimer structure of PGHS [1,2] in the model by using double concentration of the enzyme in *in silico* experiments, in comparison with the enzyme concentration in the experimental assay.

### 2.2. Consideration of NSAID Effects in the Model

We extended our models [29] to account for inhibitory effects of various types of NSAIDs, among them: irreversible/reversible, time-dependent/time-independent, selective to PGHS-1 or PGHS-2 inhibitors. 

The upper panel in Figure 1 shows the scheme of the interaction between the competitive inhibitor and the enzyme. In our model we considered NSAID binding to the unoccupied COX-site (E_1_, E_2_, E_3_, E_5_, and E_15_ enzyme states), competing with arachidonic acid. Binding of a NSAID with the COX-site was modeled by mass action reaction and characterised by two kinetic parameters: the reaction rate, *k_on_*, and dissociation, *K_d_*, constants.

In the model we assumed that NSAIDs mainly inhibit COX activity of PGHS-1 with no pronounced effect on its POX activity. In Figure 1 (upper panel) the POX_6_ cycle corresponds to the surviving POX activity of the enzyme bound with an inhibitor. The consideration of the surviving POX activity at the NSAID binding with PGHS in the model means that the enzyme bound with NSAID is incapable of catalysing COX reactions, but is still able to convert PGG_2_ to PGH_2_ in POX_6_ cycles. As it will be shown below, this effect manifests itself noticeably in PGHS-1 inhibition by NSAIDs.

Note that the surviving POX activity of PGHS-1, inhibited by NSAID, has been established experimentally [39]. Although the presence of NSAID in the COX-site is likely to influence the functioning of the POX cycle, as a result, for example, of change in Tyr385* radical formation kinetics, caused by bound NSAIDs [40,41].

### 2.3. The Method of in silico Screening of NSAID Action on PGHS-1

A kinetic model of the inhibition effect of NSAIDs on PGHS-1 was constructed in accordance with the developed catalytic cycle of PGHS-1 with consideration for the enzyme interaction with NSAID (see Figure 1) [29]. The model includes a system of 42 ordinary differential equations (ODEs), describing the dynamics of 26 enzyme catalytic states (E_i_), six metabolites (AA, O_2_, RC, OC, PGG_2_, and PGH_2_) and inhibitor binding with the enzyme [29]. To run the computational simulation and to analyse the results, we used the following software packages for kinetic modeling: DBsolve 6.1 [42] and SimBiology (MATLAB, The MathWorks Inc.). 

This model was used for *in silico* screening of the inhibition actions of different NSAIDs on PGHS-1 in *in vitro* experimental conditions. To simulate the *in silico* experiments on the NSAID interaction with PGHS-1 under *in vivo* conditions (platelets, endothelial cells), we have upgraded our model in such a way that allows taking into account the synthesis and degradation of PGHS-1 in the cells (see Figure 1) [29]. 

We also updated our model [29] to consider the reactions of PGHS-1 with external peroxide, ROOH. The reactions *V_99_ = k_7_ ∙ROOH∙E_1_*, *V_100_ = k_7_∙ROOH∙E_10_*, *V_101_ = k_7_ ∙ROOH∙E_12_*, *V_102_ = k_7_ ∙ROOH∙E_16_*, *V_103_ = k_7_ ∙ROOH∙E_18_*, and *V_104_ = k_7_∙ROOH∙E_24_* were added to the catalytic cycle of PGHS-1 (see reaction *V_99_* in Figure 1), as well to the system of ODEs. This allowed us to study the effect of external peroxide on PGHS-1 inhibition by NSAID. Note that in our simulation we used the kinetic properties of external peroxide as being the same as the peroxide PGG_2_’s properties.

### 2.4. Mechanism of PGHS-1 regulation by arachidonic acid and reducing cosubstrate

The main distinction between the *in vitro* and *in vivo* experiments on the measurement of the NSAID dose dependencies lies in the different concentrations of substrate and reducing cosubstrate in the experimental assays. In the *in vitro* experiments with the purified enzyme or a microsomal assay, high concentrations of arachidonic acid are commonly used, *i.e*., 30–100 μM [7,11,12,13], whereas in the *in vivo* experiments, the activation of platelets, endothelial cells, or blood assay is carried out by calcium ionophore or exogenous AA [9,13,14,15,16,17], and the intracellular concentration of free arachidonic acid is assumed to be less. In accordance with the estimates, the free AA concentration in platelets is in the range 0.001 μM–0.1 μM, depending on the platelet state [43,44]. 

**Figure 2:**
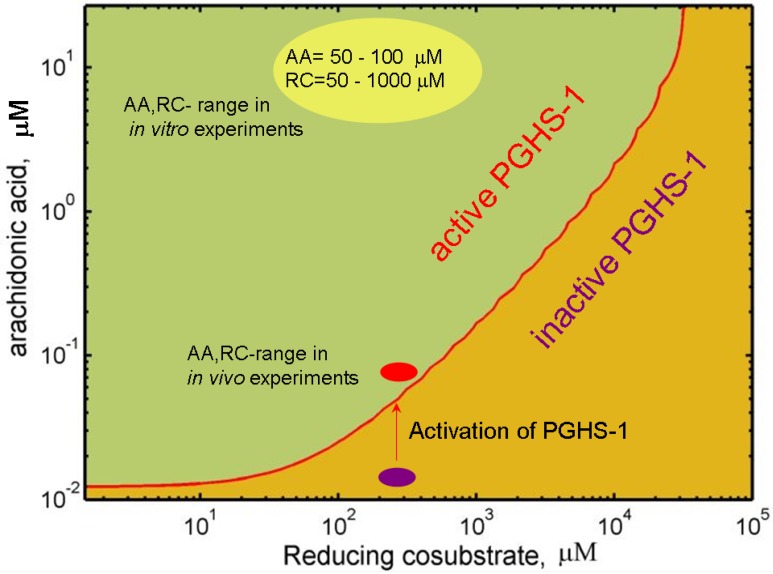
Working region of PGHS-1 depending on concentrations of arachidonic acid, AA and reducing cosubstrate, RC (phenol).

Also, the type and the concentration of the reducing substrate, RC, in *in vitro* experiments are known and fixed (phenol, tetramethyl-*p*-phenylenediamine, 50–500 μM), while in *in vivo* experiments with intact cells, the type of intracellular reducing cosubstrate and its level are unknown. 

The factors mentioned above may change the value of NSAID’s IC_50_, depending on the experimental settings. In particular, it is known from the enzymatic theory that the dependence of the competitive inhibitor IC_50_ on the substrate concentration, *S*, may be written as:

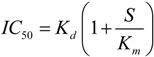
(1)
where *K_m_*—Michaelis constant for the substrate, *K_d_*—the dissociation constant of inhibitor. Equation (1) shows that less competition of inhibitor with substrate at low substrate concentration (AA) causes the NSAID IC_50_ to decrease. Thus, the increase of NSAID effect in *in vivo* conditions may be expected. It has been shown that relation (1) holds in the wide range of the substrate concentrations for PGHS-1 [29]. However, simple dependence of IC_50_ on AA concentration (1) is complicated by the complex kinetics of PGHS-1, particularly due to the existence of activation threshold in the range of low AA concentration [33]. 

Furthermore, reducing cosubstrate exerts a complex influence on the enzyme activity. Cosubstrate has been established to be an activator at low concentration and a suppressor of PGHS-1 activity at high concentrations of cosubstrate [35]. 

To study the dependence of PGHS-1 activity on AA and RC concentrations in detail, we carried out an *in silico* screening of oxygen consumption of PGHS-1 over a wide range of substrate and cosubstrate concentrations. Figure 2 shows the theoretical activation threshold curve, plotted on a plane of AA against RC concentrations. This curve divides the AA-RC plane into two areas: the enzyme is inactive in the AA and RC concentration range lying below the curve, and it is active in the upper range. According to the obtained data, the activation threshold values of AA concentration lowers to 0.01 μM in the range of RC concentration less than 10 μM, and increases up to 1 μM at RC concentration of up to 5,000 μM. Note that the obtained activation region of AA concentrations is near the estimate of the free AA concentration in platelets (0.001 μM– 0.1 μM) [44]. Below we suggest that in cellular conditions, PGHS-1 functions close to its activation threshold level (0.01 μM–1 μM).

The obtained results show that the activation threshold of PGHS-1 depends not only on the internal properties of the enzyme [45], but also on external conditions—the concentration of RC and AA. Other external factors affecting the activation threshold of PGHS-1,2 have been established to be peroxide, ROOH [33], and peroxidase levels [46]. Below we show that NSAID inhibition effect changes significantly near the activation threshold, which is determined by the AA, RC, and ROOH levels. 

## 3. *In silico* Screening of NSAID Effects at the Different Microenvironments of PGHS-1

### 3.1. In silico experiment on aspirin action on PGHS-1 under in vitro/in vivo conditions

Binding with PGHS-1, aspirin modifies the enzyme covalently by acetylation of the serine residue Ser-530 in the COX-site. This causes complete inhibition of the COX activity of PGHS-1 [1]. In our model, the irreversible PGHS-1 acetylation reaction (see reactions 69–73 in Figure 1) was modeled by action mass rate equation with a rate constant *k_on,aspirin_* =10^−5^ μM^−1^ s^−1^[29], which was estimated based on literature data [11,15,16]. Note that in the model, it is assumed that acetylated PGHS-1 retains its POX activity and is able to reduce PGG_2_ to PGH_2_ (see POX_6_ cycle in Figure 1). 

In the *in silico* experiment on aspirin inhibition of PGHS-1, we reproduced the experimental conditions of the real experiment, carried out with purified PGHS-1 [11]. In that experiment, purified PGHS-1 (0.05 μM) was preincubated for 30 minutes with different concentrations of aspirin (0–200 μM), and then substrate, AA (100 μM) and cosubstrate, RC (100 μM) were added to initiate the PGHS-1 catalysis, followed by measurement of the oxidation rate of reducing cosubstrate. Note that these values of AA and RC concentrations, corresponding to *in vitro* experimental conditions, lay far from the activation threshold of PGHS-1, obtained by us (see Figure 2). 

**Figure 3:**
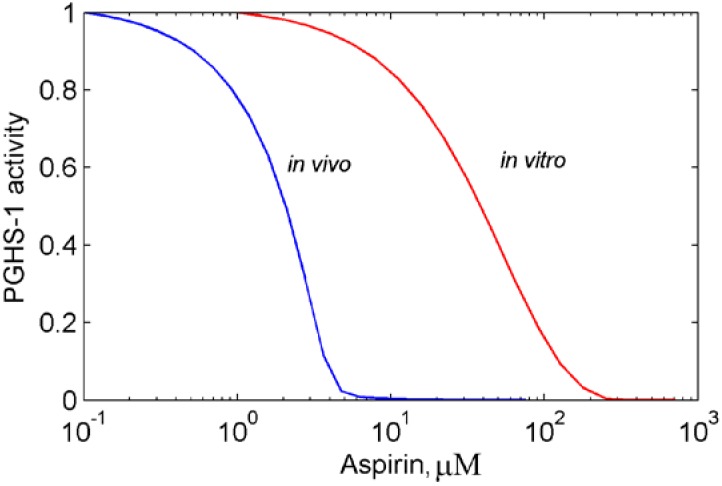
Dose dependence for aspirin calculated for purified PGHS-1 (red line) and platelet PGHS-1 (blue line). The enzyme activity (oxygen consumption (red line) and production of PGH_2_ (blue line)) is given in relative units.

According to the simulation of the dose dependence for aspirin shown in Figure 3 (red line) the value of the IC_50_ of aspirin equals 50 μM [11]. The experimentally obtained value of the aspirin IC_50_, measured in the different studies of purified PGHS-1 lays in the range of 30 μM–200 μM [13,14]. The *in silico* experiments, carried out at different experimental conditions, such as preincubation time, concentrations of AA and RC, showed that a variation of experimental settings leads to a change of the aspirin IC_50_ in the range mentioned above (results not shown).

Extensive experimental studies of aspirin effect on intact cells (platelets, endothelial cells, fibroblast, and WBA) [11,13,14,17] showed that the aspirin IC_50_, measured *in vivo* experiments, is much lower (1–3 μM), than the IC_50_ obtained in *in vitro* assays. To analyse this discrepancy, we carried out the *in silico* experiments, simulating the experiments on aspirin effect on platelets. Generally in such experiments, platelets are treated with the different concentrations of aspirin during the preincubation period of 25–30 minutes, and then the production of TxA_2_ is stimulated by calcium ionophore. The resulting concentration of TxB_2_ is then measured in 10–15 minutes [11]. 

In the *in silico* experiment under *in vivo* conditions, we adjusted our method to the simulation of PGHS-1 catalysis in the platelet environment. The following assumptions were used in the calculation: (1) There is no *de novo* synthesis of PGHS-1 in platelets (*k_syn_* = 0), but platelets contain a PGHS-1 depot. We estimated this initial concentration of PGHS-1 equal to 2.5 μM [29], based on the experimentally measured amount of TxA_2_, produced after platelet stimulation [11]. (2) PGHS-1 degradation rate, *k_deg_*, was set as 10^−5^ s^−1^ based on the experimental estimation of PGHS half-life [47]. (3) Intracellular concentration of reducing cosubstrate was assumed to be in the range of 100 μM, and its biochemical properties close to the properties of phenol, used commonly in *in vitro* experiments, as a cosubstrate.

The preincubation of platelets with aspirin and platelet activation by calcium ionophore were simulated by the following way: we assumed that in resting platelets, the concentration of AA is in the range of 0.01 μM–0.001 μM. As can be seen from Figure 2 this region lays below the activation threshold of PGHS-1 and corresponds to inactive PHGS-1. To simulate the effect of platelet stimulation by calcium ionophore, we increased AA concentration up to 0.1 μM. According to working diagram of PGHS-1 (see Figure 2), this leads to overcoming the activation threshold, and the start of PGH_2_ production. Thus we assumed that PGHS-1 functions in platelets at AA, RC concentrations close to its activation threshold (see Section 2.4 and Figure 2). Note that such a value range of AA concentrations is in accordance with the estimate of AA concentration in platelets, being in the range of 0.001 μM–0.1 μM, depending on the platelet state [44].

The resulting dose dependence curve for aspirin, acting on platelets, is presented in Figure 3 (blue line). The aspirin IC_50_ in this case is approximately 2 μM, which is in agreement with numerous experimental data obtained in experiments with washed platelets [11], and human PGHS-1 WBA [10,17].

As can be seen from the comparison of two theoretically derived dose dependencies shown in Figure 3, our model predicts a much stronger inhibitory effect of aspirin *in vivo*, as compared to *in vitro* conditions. This is indicated by a 100-fold smaller value of the aspirin IC_50_, obtained in *in vivo* experimental conditions (1 μM), as compared to the IC_50_ estimate, derived from the *in vitro* one (100 μM). An analysis of the results obtained in *in vitro* and *in vivo* conditions allowed us to draw a conclusion on the possible mechanism, underpinning the stronger inhibitory effect of aspirin *in vivo*.

**Figure 4:**
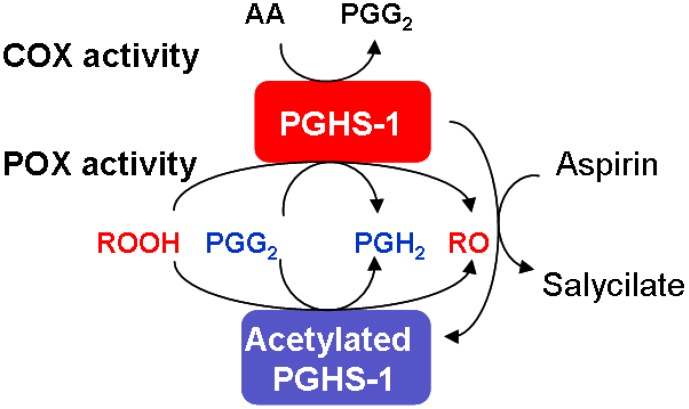
Scheme of the kinetic interaction between acetylated and non-acetylated PGHS-1 through common substrate, PGG_2_ at aspirin action. ROOH—external peroxide.

The *in silico* experiment showed that this phenomenon originates from some complex enzyme dynamics, observed in the AA-RC concentration range close to the activation threshold of PGHS-1. In this range the interaction between catalytic activities of acetylated and non-acetylated (intact) forms of the enzyme contributes significantly to the inhibition of PGHS-1 (see scheme in Figure 4). An accumulation of acetylated PGHS-1 within the cells occurs during preincubation of platelets with aspirin. As discussed in Section 2.2, the acetylated form of PGHS-1, while being inactive with regard to COX activity, can still catalyse peroxidase reaction. Residual POX activity of the acetylated enzyme leads to a fast depletion of the intermediate product, PGG_2_ from the system (see Figure 4). In this case acetylated PGHS-1 plays a role of scavenger of peroxide, PGG_2_[46]. Lack of the intermediate product disturbs PGHS-1 autocatalysis and contributes to the resulting inhibition of COX activity of the enzyme. Thus, the direct inhibitory effect of aspirin (due to acetylation of PGHS-1) is enhanced by its indirect action, resulting from scavenging intermediate product, PGG_2_ by the acetylated enzyme. Note that a kinetic interaction of acetylated and non-acetylated PGHS-1 results in a more pronounced effect on the enzyme inhibition at the concentrations of AA and RC close to the activation threshold (*in vivo* conditions), rather than far from one (*in vitro* conditions).

### 3.2. Effects of peroxide and reducing cosubstrate on PGHS-1 inhibition by aspirin

#### 3.2.1. The suppression effect of external peroxide on aspirin-mediated inhibition of PGHS-1

According to the observed effect of the interaction between acetylated and non-acetylated PGHS-1, discussed in Section 3.1, the decrease in peroxide PGG_2_ levels due to the residual POX activity of acetylated PGHS-1, leads to a rise in aspirin efficacy. Based on this observation, we suggested that the addition of peroxide to the assay may cause the opposite effect—weakening of aspirin inhibition action. To test this assumption, we updated our model to take into account the reaction of PGHS-1 with peroxide, ROOH (see Section 2.3. and Figure 1, Figure 4) and carried out *in silico* experiment on aspirin action on platelet PGHS-1 in the presence of external peroxide, ROOH.

**Figure 5:**
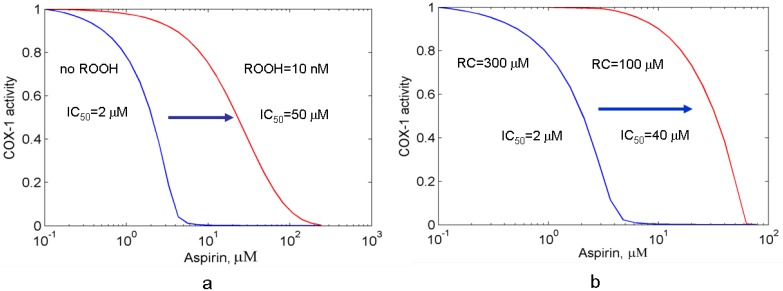
(a) The dose dependencies for aspirin calculated for PGHS-1 at the absence (blue line) and presence (red line) of external peroxide, ROOH; (b) The dose-dependences for aspirin calculated at the different cosubstrate (phenol) concentrations: 300 μM (blue line) and 100 μM (red line). PGHS-1 activity (oxygen consumption) is given in relative units.

The dose dependence of platelet PGHS-1 inhibition by aspirin, simulated in the presence of additional peroxide, is shown in Figure 5(a). As seen from the comparison of two dose dependencies, calculated in the absence/presence of external peroxide [blue and red lines, respectively, in Figure 5(a)], the addition of peroxide causes the shift of the dose dependence to the higher concentration of aspirin (red line), and increase of the aspirin IC_50_ from 2 μM to 50 μM. This result shows that external peroxide decreases the efficacy of aspirin inhibition of PGHS-1. 

In our approach this effect is explained by a weakening of the kinetic interaction between acetylated and active PGHS-1 upon the addition of external peroxide. The consumption of peroxide, PGG_2_ by acetylated PGHS-1 decreases partially due to the presence of external peroxide. It leads to acetylated PGHS-1 interfering less with active PGHS-1. Thus, external peroxide abrogates the additional inhibition effect, brought about by the interaction of acetylated and active PGHS-1 through the common metabolite, peroxide PGG_2_. 

Note that the effect of suppression of aspirin action, observed in *in silico* experiments, has been observed in *in vitro* experiments [6], in which external peroxide (12-HPETE) was obtained to decrease the aspirin-mediated inhibition of purified PGHS-1. It is also to be noted that the experimental conditions, used in the experiments [6], are close to the initial conditions in the *in silico* experiment, namely: the concentrations of ovine PGHS-1 = 1.5 μM, AA = 0.5 μM, and preincubation time equal to 30 minutes.

Moreover, experiments with cellular culture treated by aspirin have shown that the efficacy of aspirin is more than 20 times higher in A549 cells possessing a low level of hydrophobic hydroperoxides, than in RAW 264.7 cells, in which hydroperoxides are produced in quantity [6]. 

The authors of [6] proposed the hypothesis that the suppression mechanism of the aspirin-mediated inhibition of PGHS-1 by peroxide is determined by the effect of enzyme POX activity on Ser530 acetylation by aspirin. The Tyr385 residue was assumed to participate in aspirin binding with Ser530. Oxidation of Tyr385 and formation of a Tyr385 radical due to peroxide activate the POX reaction, suppressing the aspirin binding with Ser530, and thus inhibiting PGHS-1 acetylation. 

In our approach, the POX activity of the enzyme plays a key role in the suppression effect of the aspirin-mediated inhibition of PGHS-1 by external peroxide as well. But the *in silico* experiment showed that the POX activity of acetylated PGHS-1 in the presence of external peroxide affects the inhibition of active PGHS-1 less, than in the case of the absence of external peroxide (see discussion in Section 3.1). Thus, the addition of external peroxide leads to the decrease in suppression action of the POX activity of acetylated PGHS-1 on the non-acetylated enzyme. 

The obtained results lead one to conclude that different peroxide status of different cells, as well as a change in peroxide level of cells, as a result of diseases, may alter the aspirin efficacy and cause aspirin resistance [28].

#### 3.2.2. Effect of reducing cosubstrate on the aspirin-mediated inhibition of PGHS-1

In Section 2.4 we showed that the reducing cosubstrate substantially influences the activation threshold of PGHS-1 (see working diagram in Figure 2). The activity of the enzyme drops dramatically near the threshold region of cosubstrate concentrations. Note that the threshold RC concentration for PGHS-1 has been established experimentally [35]. As we suggested, PGHS-1 functions in a cell at the cosubstrate concentrations near its activation threshold (see Figure 2). Further we study the effect of RC on inhibition effect of aspirin in the RC concentration range near the activation threshold. 

**Figure 6:**
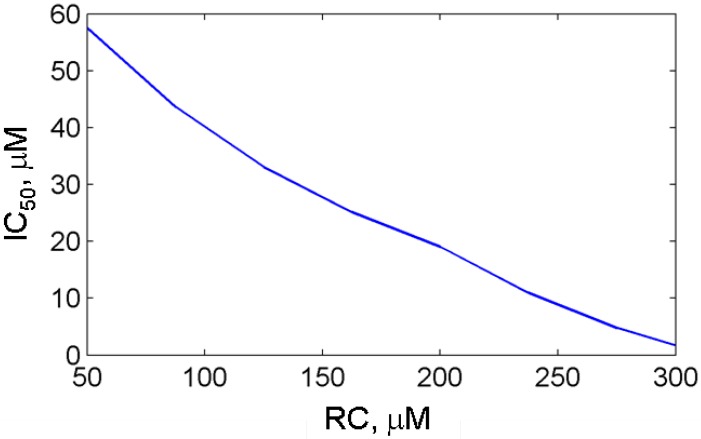
The dependence of the aspirin IC_50_ on cosubstrate (phenol) concentration, RC calculated for purified PGHS-1.

Figure 5(b) shows the theoretical dose dependencies for aspirin calculated at RC concentrations, 300 μM and 100 μM (blue and red lines, respectively). As it can be seen from the working diagram in Figure 2, the first simulation was carried out at the concentration value closer to the activation threshold (AA = 0.1 μM, RC = 300 μM) than the second one (AA = 0.1 μM, RC = 100 μM). The comparison of two dose dependencies, given in Figure 5(b) shows that moving of AA,RC-region far from the activation threshold leads to the shift in the dose dependence to the higher concentrations of aspirin, and an increase in its IC_50_ from 2 μM up to 40 μM. These results of the *in silico* screening of the aspirin IC_50_ show that the efficacy of aspirin falls off with RC concentration (see Figure 6). It is to be mentioned that the most pronounced effect of RC on the aspirin IC_50_ is observed in the RC concentration region, close to the activation threshold of PGHS-1.

### 3.3. In silico screening of reversible inhibitor actions in different environmental conditions of PGHS-1

We applied the developed method to *in silico* experiments with reversible PGHS-1 inhibitors, such as celecoxib and ibuprofen. Celecoxib is a reversible, time-independent, selective inhibitor of PGHS-2, with selectivity equal to 0.1–0.7 (ratio of IC_50_ of PGHS-2 to IC_50_ of PGHS-1) [7,9,18], and ibuprofen is a reversible, time-independent, selective inhibitor of PGHS-1 with a selectivity of 0.67–53.3 [10]. 

**Figure 7:**
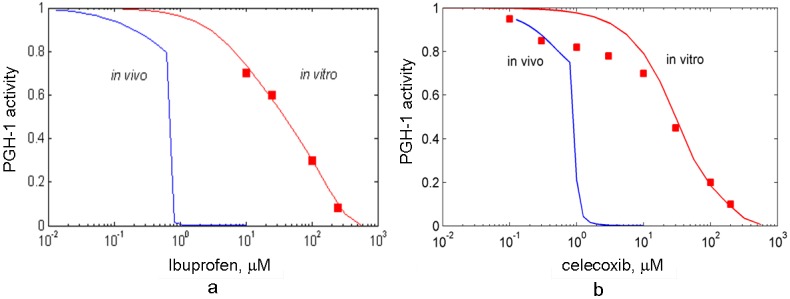
The dose dependencies of PGHS-1 inhibition by celecoxib (a) and ibuprofen (b) calculated for purified (red line) and platelet PGHS-1 (blue line). Points—experimental data for purified PGHS-1 [7]. The enzyme activity (oxygen consumption (a) and production of PGH_2_ (b)) is given in relative units.

To estimate the affinities of these drugs to PGHS-1, we reproduced *in silico* the *in vitro* experiments on the inhibitory actions of celecoxib and ibuprofen on purified PGHS-1 [7], and obtained their kinetic parameters as a result of the fitting of the experimental and simulation dose dependencies for celecoxib and ibuprofen. We obtained the following values of the reaction rate and dissociation constants of celecoxib: *k_on,celecox_* = 1.6 μM^−1^ s^−1^, *K_d,celecox_* = 0.2 μM and ibuprofen: *k_on,ibuprofen_* = 7 μM^−1^ s^−1^, *K_d,ibuprofen_* = 0.1 μM. Both the theoretical and experimental [7] dose dependence curves for celecoxib and ibuprofen are presented in Figure 7. The calculated IC_50_ of celecoxib and ibuprofen are in the range of 30 μM. The experimental values of the IC_50_ of ibuprofen lay in the wide range of the concentrations, 3.3 μM–100 μM [7,13,48,49,50]. 

We applied our technique to translate the results obtained in the *in vitro* conditions to the *in vivo* ones. The *in silico* screening of the drug action on platelet PGHS-1 was carried out at the concentrations of RC and AA near the activation threshold of the enzyme (see Figure 2). The details of the experimental procedure and computer simulation are the same as in the case of the *in silico* study of aspirin action on platelets, described in Section 3.1. 

The calculated dose dependencies for celecoxib and ibuprofen are shown in Figure 7. Similarly to the aspirin case (see Section 3.1), celecoxib and ibuprofen demonstrate substantially more potent effects in *in silico* experiment with the *in vivo* experimental conditions than in *in vitro* settings. We established two key mechanisms of the changes in celecoxib and ibuprofen inhibitory effect in *in vivo* conditions. The first one is due to the additional suppression effect, caused by the interference between the residual POX activity of PGHS-1 bound with inhibitor and active PGHS-1. This mechanism, discussed in Section 3.2, manifests itself near the activation threshold of the enzyme, and causes the shift and shape change of the dose dependence.

The second reason for the decrease in IC_50_ is the concentration of the substrate, AA, being lower in *in vivo* experiments compared to in *in vitro* ones. As celecoxib and ibuprofen are fast, reversible inhibitors of PGHS-1, competing with the substrate, the dependencies of their IC_50_s on the substrate concentration may be approximately described by Equation (1). According to this dependence, the fall in the AA concentration leads to the decrease in the IC_50_ value. Note that this mechanism, caused by the drug and AA competition, does not show up in the case of *in vitro* and *in vivo* experiments with platelets treated by slow irreversible inhibitor, aspirin. This effect is due to the fact that enzyme binds with aspirin in the absence of competitive substrate at the preincubation stage.

The obtained value of the celecoxib IC_50_ (1 μM) is in agreement with the experimental values [9,11,18], which vary in the range from 1.2 μM to 10 μM [9,11,18]. The observed differences in the reported values of the celecoxib IC_50_ are most likely to be explained by the variations in the experimental conditions used in different studies. For example, some experiments were performed on washed platelets [11], whereas the others—on human PGHS-1 WBA [9]. The methods of platelet activation were different as well: stimulation by exogenous AA, or calcium ionophore. Note that platelet activation methods have been reported to influence significantly the IC_50_ value [50].

The results of our calculations also showed that ibuprofen produces a stronger inhibitory effect on PGHS-1 *in vivo* condition than *in vitro* one. The obtained value of the ibuprofen IC_50_ is 2 μM, which is in agreement with the experimental data, measured in the different experimental conditions: 7.6 μM (WBA) [9], 1.4 μM–0.4 μM (washed platelets) [11], 5 μM (bovine aortic endothelial cells) [13]. 

The *in silico* screening of celecoxib and ibuprofen actions on PGHS-1 showed that these two drugs with different selectivity to PGHS-1 possess approximately equal abilities to inhibit PGHS-1 in platelets and in *in vitro* conditions. Below we continue to compare these NSAIDs by using *in silico* experiments in the different experimental conditions.

### 3.4. In silico screening of combined effects of NSAIDs on PGHS-1 inhibition in vitro/in vivo conditions

The problem of NSAID co-administration is being actively discussed in literature in the context of uncertainty of the resulting therapeutic and side effects which arise from such combinations [11,26,27]. Both *in vitro* and *in vivo* experiments, as well as clinical studies, demonstrate that some nonselective NSAIDs (ibuprofen, naproxen) [11,26] and PGHS-2 selective NSAIDs (celecoxib, valdecoxib, etoricoxib) [11] can undermine the aspirin-mediated inhibition of purified and cellular PGHS-1. That suppression effect is assumed to be caused by the interference of aspirin and some NSAIDs upon their competitive binding with PGHS-1 [28]. But, the detailed mechanism of such suppression has been difficult to explain satisfactorily. 

To elucidate the mechanism of the interference of NSAIDs, we adapted our method to the *in silico* screening of the resulting effect of two NSAIDs in combination on PGHS-1 *in vivo* and *in vitro* conditions. In this section, we demonstrate the application of our method to *in silico* study of the combined effects of aspirin and ibuprofen, as well as aspirin and celecoxib. 

**Figure 8:**
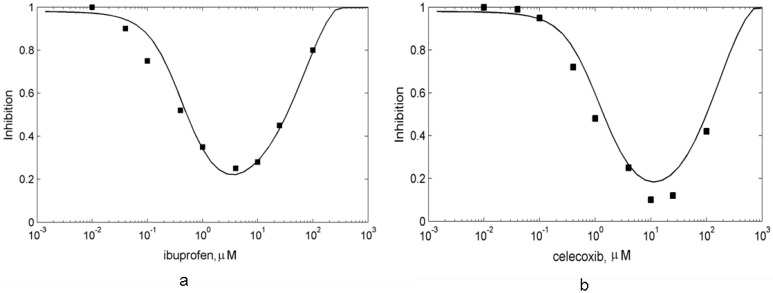
Effect of the suppression of aspirin-mediated inhibition of purified PGHS-1 by ibuprofen and celecoxib. The dose dependence of PGHS-1 inhibition by ibuprofen (a) and celecoxib (b) at the presence of 200 μM aspirin. Lines—theoretical results, points—experimental data [8]. Inhibition of oxygen consumption by PGHS-1 is given in relative units.

In *in silico* experiments we reproduced the conditions of the experiments on a combined action of aspirin with celecoxib, and aspirin with ibuprofen on PGHS-1 *in vitro* conditions and platelets [11]. In the experimental study, purified PGHS-1 was preincubated with 200 μM aspirin and different concentrations of ibuprofen (celecoxib) for 30 minutes [11]. Cyclooxygenase reaction was then initiated by the addition of 100 μM of arachidonic acid and 100 μM of reducing cosubstrate (tetramethyl-*p*-phenylenediamine), and the rate of cosubstrate oxidation was monitored spectrophotometrically [11]. The experimentally measured dose dependencies for ibuprofen (celecoxib) are presented in Figure 8 (points). In the *in silico* experiment we used kinetic parameters of aspirin, ibuprofen, and celecoxib identified previously (see Section 3.3). 

The obtained theoretical dose dependencies for ibuprofen and celecoxib in the presence of 200 μM aspirin are shown in Figure 8 (solid lines). As it can be seen, the calculated curves provide a good agreement with the experimental data [11], shown by points in Figure 8. The resulting dose dependencies demonstrated that ibuprofen and celecoxib suppress the inhibitory effect of aspirin by 80% and 90%, respectively, in the drug concentration range of 0.2 μM–100 μM. The minimum of inhibition is reached at approximately 4 μM for ibuprofen and 10 μM for celecoxib. The increase of drug concentrations leads to a full recovery of the inhibitory effect of the drug combination.

In the framework of our model, the observed suppression effect of the aspirin-mediated inhibition of PGHS-1 by ibuprofen or celecoxib may be explained by the complex kinetic interplay of three species—aspirin, ibuprofen (celecoxib) and arachidonic acid—all competing for the same COX binding site of the enzyme. These three small molecules differ by their kinetic properties—the rate of binding and the affinity to the COX-site of the enzyme, which results in an antagonistic effect between these species in the wide range of their concentrations. As has been established experimentally [8] and theoretically, 200 μM of aspirin is enough to completely acetylate 0.5 μM of PGHS-1 in the end of 30 minutes preincubation period [see the initial point on the dose dependence curve at zero concentration of ibuprofen (celecoxib) in Figure 8]. Addition of ibuprofen (celecoxib) at the preincubation stage results in the competitive binding of two inhibitors with PGHS-1 which leads to the formation of two enzyme states: acetylated PGHS-1 and PGHS-1 bound with ibuprofen (celecoxib). The relation among the concentrations of these enzyme states and the free enzyme is defined by the preincubation time and the kinetic properties of the drugs. In general, ibuprofen (celecoxib) binds with PGHS-1 (*k_on,ibuprofen_* = 7 μM^−1^ s^−1^; *k_on,celecox_* = 1.6 μM^−1^ s^−1^) faster than aspirin (*k_on,aspirin_* = 10^−5^ μM^−1^s^−1^), and therefore tends to replace aspirin molecule in the COX-site at the high concentration of ibuprofen (celecoxib). It leads to saving some part of PGHS-1 from irreversible acetylation by aspirin. The substitution of aspirin by ibuprofen (celecoxib) starts when the drug concentration is in the range of the dissociation constants of ibuprofen, *K_d,ibuprofen_* = 0.1 μM or celecoxib, *K_d,celecox_* = 0.2 μM (see Section 3.2). The simulation of the preincubation stage showed that at 10 μM of celecoxib, 90% of PGHS-1 are bound with celecoxib, while the rest 10% are bound with aspirin. Note that in the end of the preincubation period (30 minutes), all PGHS-1 are totally inhibited by aspirin and ibuprofen (celecoxib). When the enzyme substrate AA is added, it gets involved in the antagonistic interplay with ibuprofen (celecoxib) for the COX-site. As our calculations have shown, the dissociation constant of ibuprofen (celecoxib) is close to the one of AA (*K_d,AA_* = 0.1 μM [29]). Considering this and the fact that the concentration of AA in the experiment equals to 100 μM [11], AA intensively replaces ibuprofen (celecoxib) in the COX-site of the enzyme, and activates it at low ibuprofen (celecoxib) concentrations (0.1 μM–1 μM). It results in the inhibition dose dependence falling in the range of ibuprofen (celecoxib) concentrations 0.1 μM–1 μM (see Figure 8).

The rise in the inhibition dose dependence curve observed in the range of 10 μM–100 μM of ibuprofen (celecoxib) concentrations is determined by the inhibition action of ibuprofen (celecoxib) in the region of its IC_50_ (≅30 μM). 

**Figure 9:**
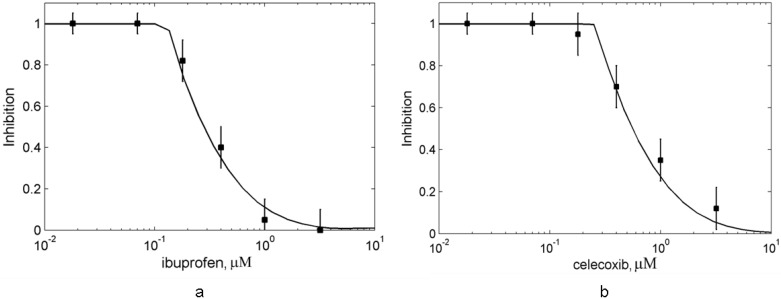
Effect of the suppression of aspirin-mediated inhibition of platelet PGHS-1 by ibuprofen and celecoxib. The dependence of platelet PGHS-1 inhibition on ibuprofen (a) and celecoxib (b) concentrations at the presence of 100 μM aspirin at the full removal of ibuprofen (celecoxib) after preincubation. Lines—theoretical results on the inhibition of PGH_2_ production, points—experimental data on the inhibition of TXB_2_ production [8].

The suppression effect of ibuprofen (celecoxib) on the aspirin-mediated inhibition of PGHS-1 has been confirmed in experiments on platelets [11] (see experimental data in Figure 9). To analyse the difference in these effects in the cases of *in vitro* and *in vivo* experimental conditions, we carried out a computational simulation of the combined action of aspirin and ibuprofen (celecoxib) using the experimental conditions of the work [11]. In this experiment, platelets were treated with 0–100 μM ibuprofen (celecoxib) for five minutes before the addition of 100 μM aspirin. After 20 minutes incubation, the platelets were washed to remove the reversible inhibitor, and then activated by calcium ionophore. The amount of TXB_2_ produced was measured after 10 min of the cyclooxygenase reaction. In the *in silico* experiment the intracellular concentration of AA and RC were chosen near the activation threshold of PGHS-1 (see discussion in Section 3.1 and Figure 2).

The theoretical dose dependencies for ibuprofen and celecoxib in the presence of 100 μM aspirin are shown in Figure 9 along with the experimental data [11]. These results show that the blocking effect of ibuprofen (celecoxib) on the aspirin-mediated inhibition of PGHS-1, observed on the purified enzyme, is maintained in *in vivo* case as well. However, there is the noticeable difference between these two cases. There is no restoration of the initial inhibition level in the range of ibuprofen (celecoxib) concentrations higher than 10 μM. The activation of platelets at these drug concentrations is determined by complete removal of ibuprofen (celecoxib) from intercellular environment of platelets [11]. 

**Figure 10:**
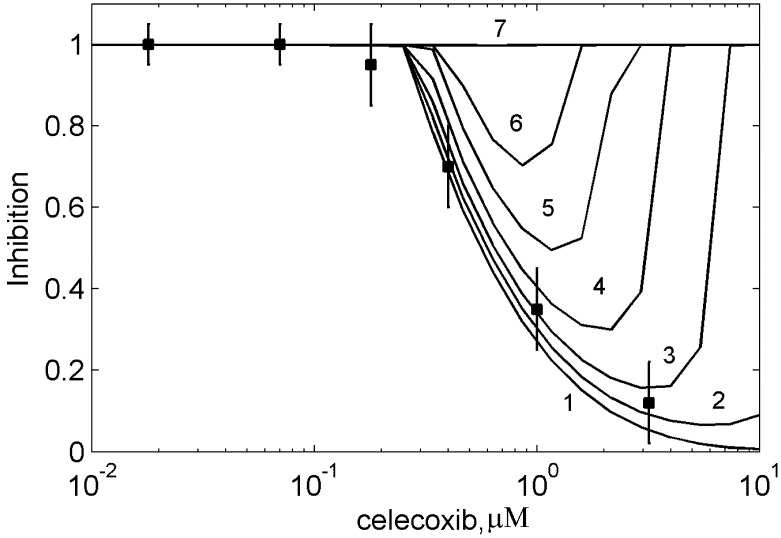
Effect of the suppression of aspirin-mediated inhibition of platelet PGHS-1 by celecoxib. The theoretical dependencies of platelet PGHS-1 inhibition on celecoxib concentration calculated at the partial removal of celecoxib after preincubation. Line 1—100% removal of celecoxib, line 2—70%, line 3—50%, line 4—30%, line 5—20%, line 6—10%, line 7—no removal of celecoxib. Lines—theoretical results on inhibition of PGH_2_ production, points—experimental data on inhibition of TXB_2_ production by platelets washed after preincubation [8]. Inhibition is given in relative units. Aspirin concentration—100 μM.

To estimate the impact of intercellular concentration of the drug on the blocking effect, we repeated the *in silico* experiment under the conditions of partial removal of celecoxib after the preincubation stage. The resulting dose dependence, given in Figure 10, showed the disappearance of the dip on the dose dependence curve in the range of 1 μM celecoxib concentration when the residual concentration of celecoxib is rising. In contrast to *in vitro* case (see Figure 8), the blocking effect vanishes at the high concentrations of celecoxib in platelets (see line 7 in Figure 10). The dip disappearance on the dose dependence curve calculated for *in vivo* conditions is determined by the difference in the celecoxib IC_50_ obtained in *in vitro* and *in vivo* cases. As we have established above, a rise in the dose dependence at high drug concentrations is governed by the value of the celecoxib IC_50_. 

Under *in vivo* conditions, the celecoxib IC_50_ (1 μM) is less than in *in vitro* case (30 μM) and close to the minimum of the dose dependence curve (at 1 μM). In this case the dip in the dose dependence curve is not formed. Note that the same effect of the disappearance of the blocking effect was observed in the case of the combination of aspirin and ibuprofen (data not shown). Thus, the *in silico* experiments showed that blocking effects of ibuprofen and celecoxib on the aspirin-mediated inhibition of platelet PGHS-1 depend significantly on the drug concentration in the intercellular environment. So the further *in silico* study of the conditions, at which NSAIDs block aspirin inhibitory effect on PGHS-1 in platelets, needs the consideration of pharmacokinetic data.

## 4. Conclusions

The developed method of the *in silico* experiments on NSAID action on PGHS-1 was shown to be a promising tool to be applied to the following problems of *in silico* pharmacology:

- *In silico* screening of inhibition effects of different types of NSAIDs on PGHS-1 in various environmental conditions of the enzyme;
- Dissection of the key factors, which determine the variability of PGHS-1 response to NSAID action in *in vitro* assays, platelets, endothelia and other cells;
- *In silico* comparison of the different NSAIDs and the establishment of bioequivalence of the various drugs in the different experimental conditions;
- Translation of the results on NSAIDs action on PGHS-1, obtained in *in vitro* experimental conditions to *in vivo* settings;
- *In silico* study of the drug interference, underlying the combined action of two NSAIDs on PGHS-1 to predict the potential risks and benefits of NSAID combination therapy.

The proposed method of the *in silico* screening of PGHS-1 inhibition by NSAIDs in the intracellular environments corresponding to the inflammation progression in different cells may be a useful technique for predicting the anti-inflammatory and adverse effects of NSAIDs and their combinations in the changeable intracellular conditions. 
